# Testing of NKA expression by mobile real time PCR is an efficient indicator of smoltification status of farmed Atlantic salmon

**DOI:** 10.1016/j.aquaculture.2021.737085

**Published:** 2021-11-15

**Authors:** Michael McGowan, Simon MacKenzie, Nikos Steiropoulos, Manfred Weidmann

**Affiliations:** a Institute of Aquaculture, University of Stirling, Stirling, United Kingdom; b Europharma Scotland Glasgow, United Kingdom; c Medizinische Hochschule Brandenburg Theodor Fontane, Senftenberg, Germany

**Keywords:** Sodium potassium ATPase, Smoltification, ATPase (NKA) α1a mRNA, Salmon, NKA activity

## Abstract

Assessment of seawater readiness of freshwater salmon smolts is a crucial husbandry step with economic implications in salmon aquaculture but current methods rely on delayed centralised enzymic activity measurement. The efficiency of a qRT-PCR assay for sodium potassium ATPase (NKA) α1a mRNA was tested in a 3-year study on 19 hatcheries across Scotland incorporating environmental factors such as temperature and metal contamination. The NKA qRT-PCR assay was transferred to a mobile laboratory and on-site testing was carried out at 3 hatchery sites. For the first two years standard enzymatic and gene expression assays had similar success rates in detecting smoltification (NKA activity 60%, qRT-PCR 57%). In the third year, all but one site were determined as sea water ready by qRT-PCR but only at 4 by enzymatic testing. On site testing with mobile qRT-PCR was successfully performed on four farm sites. Altogether, high sensitivity was shown for the in lab (98.9%, SE 0.24) and mobile (93.43%, SE 0.119) assays when tested using a quantitative RNA standard. Some indication for obscured smoltification assay results due to environmental increased heavy metal contamination was observed.

Our results prove it is possible to test a smoltification marker on site and provide results on the day of testing during the smolt period allowing for informed decisions on seawater transfer.

## Highlights

• A qRT-PCR assay for sodium potassium ATPase (NKA) α1a mRNA is efficient for the determination of smoltification status of smolts.
• The qRT-PCR assay can be performed in a mobile laboratory on - site time to results is about 4–5 h.
• On farm use ca speed up reliable determination of sewater readiness.

## 1 Introduction

Atlantic salmon (*Salmon salar*) exhibit an anadromous lifecycle, where hatching and early development are confined to fresh water (FW), followed by a migratory phase into salt water (SW) and significant growth before completing their adult lifecycle by returning to spawn in FW (Folmar and Dickhoff, 1980; Hoar, 1976). This life cycle requires major physiological osmotic adaptation in juveniles and seasonal photoperiod and temperature initiate this smoltification process (Duston and Saunders, 1990; McCormick et al., 2000; Bjornsson and Bradley, 2007; Handeland et al., 2013).

Driven by the endocrine system salmon parr adapt into SW ready smolts through a number of morphological, physiological, behavioural and biochemical changes (Hoar, 1976; Folmar and Dickhoff, 1980; Barron, 1986; McCormick and Saunders, 1987; Dickhoff et al., 1997; McCormick, 2001; Ebbesson et al., 2008; McCormick et al., 2009, Stefansson et al., 2012). Studies on endocrine factors concluded that growth hormones (GH), thyroid hormone (Prunet et al., 1989; McCormick, 1995, McCormick, 1996, McCormick, 2001, McCormick, 2013; Agustsson et al., 2001; Ebbesson et al., 2008; Bjornsson et al., 2011), insulin like growth factor (IGF—I) (McCormick, 1995, McCormick, 1996, McCormick, 2001; Dickhoff et al., 1997; Agustsson et al., 2001) and cortisol (McCormick, 1996, McCormick, 2001; McCormick et al., 2000, Kiilerich et al., 2007, Ebbesson et al., 2008) play a significant role in osmoregulation in salmon by inducing development of iononcytes in the gill epithelia switching the function from an ion absorbing to an ion secreting role. McCormick and Saunders (1987) suggested that the transport protein Sodium Potassium ATPase (NKA) is responsible for salt regulation and seawater acclimation in salmon.

NKA concentration has been shown to consistently increase in the gills during smolt SW acclimation (Prunet et al., 1989; McCormick, 1995, McCormick, 1996, McCormick, 2001; 2000; Bystriansky and Schulte, 2011; Handeland et al., 2013) and acts in conjunction with the Na(+)/K(+)/2Cl(−) cotransporter (NKCC), which assists in salt secretion through gill chloride cells (Pelis et al., 2001), and the cystic fibrosis transmembrane regulator (CFTR) which is an apical Cl- channel functioning under favourable electrical gradients (Marshall and Singer, 2002). NKA is composed of two major subunits alpha and beta, each having several isoforms. Of the five NKA alpha isoforms (α1a, α1b, α1c α2a and α3), α1a expression decreases whilst α1b increases when Atlantic salmon are transferred to SW (Mackie et al., 2005; Bystriansky et al., 2006; Nilsen et al., 2007; Madsen et al., 2009). Further data on exposure of mature SW living salmon to FW showed a 7-fold increase in expression of α1a and 60% decrease in α1b. This trend was also confirmed in wild salmon and several additional studies indicate that α1a is the FW isoform and α1b the SW isoform of NKA in Atlantic salmon (Bystriansky and Schulte, 2011, Stefansson et al., 2012), McCormick et al., 2009 and Madsen et al., 2009). Recent research into smolitifcation biomarkers further supports this as salmon with high α1a activity show increased mortality rates in SW (Houde et al., 2019a; Houde et al., 2019b). Clearly both isoforms have an important role in SW regulation and acclimation and are candidate biomarkers for monitoring smoltification in salmon.

Currently the most commonly used methods for smoltification assessment in Atlantic salmon are the smolt index, SW bath tests, plasma chloride measurements and the ATPase enzymatic activity assay (NKA activity assay). These measures are used individually or in combination to support management decisions in salmon aquaculture regarding timing of SW transfers. The smolt index is based upon visible morphological changes; however, it is limited in its approach due to the subjective non-quantitative 3 point scoring system that is dependent upon user interpretation. SW bath tests were the main standard test used to assess seawater readiness before the development of NKA activity assays however SW baths are based on survival rate and therefore present an unacceptable welfare issue due to extended suffering. Plasma chloride assessment has been used as changes in plasma chloride concentration closely mirror the osmotic change in Atlantic salmon (Solbakken et al., 1994). Outcomes are based upon the ability of SW ready smolts to maintain a stable concentration (150 Cl mM) of plasma Cl^−^ through osmoregulatory acclimation to SW. This test also suffers from similar welfare issues stated in the SW bath tests above requiring prolonged stressful and harmful procedures. Ion regulatory tests based upon ion chromatography methods (often for Na^+^, K^+^ and Cl^−^) have also been used to assess the SW readiness of smolts although have not to date proven robust enough for the wider uptake of these methods.

Gill based NKA activity assays were developed to focus on the measurement of gill NKA activity using salmon gill tissue throughout the smolt period (Zaugg, 1982). The gill arch is removed and NKA enzyme activity is directly measured in a classical inhibitor-based ATPase enzyme activity assay. NKA activities obtained are expressed in μmol ADP/mg protein^−1^ h^−^. A NKA activity threshold is then used to determine if smoltification has occurred.

NKA activity assays are currently regarded as a reliable indicator of smoltification due to the tests being objective and quantifiable. However, due to the different roles α1a and α1b play in SW acclimatization this assay may not provide a completely accurate picture on the overall role of the protein (Richards et al., 2003). In addition, data robustness has been questioned due to the inherently high variation in results obtained. Nevertheless, NKA activity assays have been widely accepted and are applied through centralised laboratory services on an industrial scale. The technique however is costly, time consuming and not suitable for field use.

Surprisingly, very few developments using qRT-PCR assays utilizing potential biomarkers within salmon during smoltification have been described (Olsvik et al., 2005). Due to the recent development of mobile qRT-PCR platforms and the ease, speed and relative cheapness of the technique, a fieldable assay to successfully track and detect the point of optimal SW transfer of Atlantic salmon should be highly desirable for the industry. With numerous indicators of smoltification available (T-GH, GH, cortisol and IGF-1), testing and development of qRT-PCR assays could provide a fast and effective technique for smoltification analysis. In this study we focused on the development of a novel NKA qRT-PCR assay for NKA α1a mRNA. We conducted an initial 3-year study on active hatcheries across Scotland to compare the efficiency of our NKA qRT-PCR assay with the industry standard NKA activity assay at detecting smoltification. Furthermore, some sites used Supersmolt® feed, a commercial diet from STIM Scotland Ltd., that promotes and stimulates hypo-osmoregulatory changes in salmon during the smolt window. This aims to promote a higher percentage of the population to enter the SW transfer window simultaneously thus reducing variation across the population. In this study S0 fish were fed with Supersmolt® for a period of 4–6 weeks under 24 h light. During this study we also developed a mobile qRT-PCR platform for NKA α1a and conducted field tests onsite at hatcheries.

## 2 Materials and methods

### 2.1 Fish handling and sampling

**Fig. 1:**
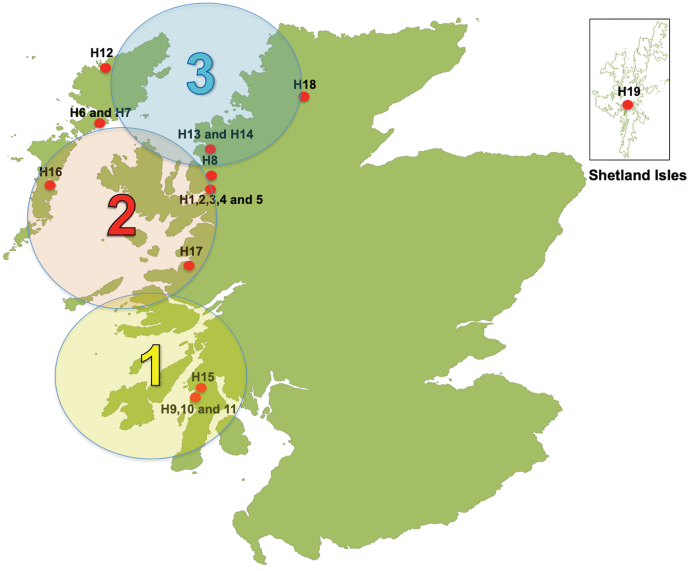
Map indicating 19 hatcheries sampled and temperature profile locations.

Gill tissue samples were collected from 19 hatcheries across Scotland over a 3-year sampling period (2015–2017). All farms were located on the Western Coast of Scotland and used FW tanks for smolt production, except for hatchery (H) 18, which used FW cages. The farms were located from the Mid Western Coast to the North Western coast and across to the Isle of Harris, with the most Northern farm located on the Shetland Islands (H19) (Fig. 1.). The farms varied in size and production capacity with H1–5 and H9–11 the largest producers, and H12 and H16 with significantly lower production.

Some sites used Supersmolt® feed, a commercial diet from STIM Scotland Ltd., that promotes and stimulates hypo-osmoregulatory changes in salmon during the smolt window. This aims to promote a higher percentage of the population to enter the SW transfer window simultaneously thus reducing variation across the population. In this study S0 fish were fed with Supersmolt® for a period of 4–6 weeks under 24 h light.

For each year's analysis the secondary gill arch was collected at 16 hatcheries (2015), 13 hatcheries (2016) and 9 hatcheries (2017) for NKA activity assay (*n* = 25) and NKA qRT-PCR (*n* = 8) from S0 smolts. Both left and right gill arches were collected from the same fish at each time point for NKA qRT-PCR and NKA activity analysis. Gill samples for NKA activity analysis were collected in 1.5 ml tubes containing a 1% Sucrose-EDTA imidazole deoxycholate (SEID) solution, and for NKA qRT-PCR analysis in 1.5 ml tubes containing RNA later. Initial (I), mid (M) and final (F) time point were pre-determined with a window of 1–3 weeks between each point. I-points corresponded with the beginning of feeding of salmon with SuperSmolt® (STIM Scotland Ltd.) only, and F-points with the ending of feeding before SW transfer. M-points were taken between 2 and 3 weeks after initial feeding (Supplement tables ST1-ST6). A constant light photoperiod regime (24 h) was utilised throughout the testing period (I – F points) at each site over the 3-year study. Analysis of NKA enzyme activity assays were conducted at the lab at STIM Scotland Ltd. NKA qRT-PCR analysis were carried out at the Institute of Aquaculture, University of Stirling. Sites sampled, as well as number of tanks varied from year to year with some only being sampled over 1 or 2 years.

### 2.2 NKA enzyme activity assay

ADP standards were checked everyday to ensure measured conversion of NADH to NAD+ was within a kinetic correlation coefficient of >0.99% with an optical density (OD) of between 0.6 and 0.8 between the time points of 0–150 s. The ADP standard curve used ranged from 1 nM - 25 nM. ADP standards were defrosted, vortexed and a 10 μl volume pipetted into a 96 well flat bottom non-sterile ELISA plate in duplicate for each value range. 200 μl of Na^+^ K^+^ ATPase inhibitor –ve ouabain was added to each well and ran in a Versa max microplate reader (SoftMax 4.5.4) for 5 mins at 25 °C. If the standards were outside the acceptable range the plates were re-run and/or new standards of –ve ouabain were made. For each day of analysis, three concentrations of SEID solution were made (0.5%, 1% and 7:3) along with an assay mix containing 0.23 nm NADH, 1 M imidazole, salt solution, 0.53 mM adenosine 5’triphosphate disodium triphosphate (ATP), 2.1 mM phosphoenolpyruvate monopotassium salt (PEP), b-nicotinamide adenine dinucleotide reduced disodium salt hydrate (NADH), 3.75 mM L-lactate dehydrogenase from rabbit muscle, (LDH) pyruvate kinase from rabbit muscle (PK) and distilled water (dH_2_0). Twenty-five gill samples from farms and 2 quality control gill samples of known activity were prepared per run. Once defrosted, gills were placed in a 5 ml tissue grinder tube containing 1 ml of 7:3 SEID solution. Gills were then manually crushed for 1 min. The gills were graded against a dilution factor based on fish weight, and volumes of gill homogenate transferred into 1.5 ml tubes in the corresponding volume of 0.1% SEID solution. All samples were then centrifuged at 350*g* for 5 min and the supernatants transferred to 1.5 ml tubes and placed on ice. Bradford protein assays were performed at 25 °C for 5 min on all samples to determine if the proteins fell between a range of 500-1500μg/ml. If proteins fell outside this range the dilution of supernatant to H_2_O was recalculated. 80 μl of each sample were transferred into a new 1.5 ml tube containing 320 μl of H_2_O. 10 μl of each sample and a protein standard curve were pipetted onto a 96 well plate in quadruplicate and 200 μl of –ve ouabain was then added to each well and analysed. Once the protein analysis was verified, 10 μl of each sample was pipetted into a 96 well plate in octuplicate. 200 μl of –ve ouabain was added to one half of the plate containing 4 of the 8 repeated samples, with 200 μl of +ve ouabain added to the other half. NKA activity assay analysis was then performed at 25 °C for 20 min. NADH disappearance was measured at 340 nm and NKA activity (μmol ADP mg protein^−1^ h^−1^) generated based on the variance between the absence and presence of ouabain on ATP hydrolysis. A threshold for determining if smoltification had occurred was used at an activity level of 10 μmol ADP/mg protein^−1^ h^−1^ and above for the protein. This threshold is based on previous successful transfer data obtained from STIM Scotland Ltd. over several years. Known quantitated samples from the previous S0 season were used as quality controls for both the NKA activity assay and protein readings alongside the unknown samples.

### 2.3 RNA extraction

RNA extractions were carried out using the QuickGene Mini80 extraction robot using the RNA tissue kit II (RT-S2) extaction kit according to the manufacturer's instructions (Kurabo, Japan). Briefly, 10–20 mg of gill tissue was placed in 1.5 ml tubes containing 500 μl of lysis buffer, three 3.5 mm glass beads and 0.1 mm zirconia beads, vortexed for 30s followed by centrifugation for 3 min at 16800*g* in a 5418R centrifuge (Eppendorf) twice. For each sample 350 μl supernatant was transferred to a new 1.5 ml tube and 175 μl of solubilisation buffer added and briefly vortexed. Following this 175 μl of ethanol (70%) was added to each sample, the mixture vortexed for 1 min and briefly spun down in the centrifuge. The supernatants of eight samples were transferred into the individual microtube columns of the Mini80QuickGene cartridge. The supernatant was washed through the column followed by 3 wash steps with 750 μl of wash buffer for each sample. To ensure high RNA yield 50 μl of elution buffer was added to elute for 4 min before being washed through into empty 1.5 ml tubes. RNA concentration was determined using a spectrophotometer (NanoDrop-1000, (software version 3.8.1) Thermo Scientific). Extracted RNA was then diluted (200-400 ng/μl) and stored until use at −70 °C.

### 2.4 Dried standard, primers and probe

For optimal long term storage and stability, DNAstable® plus (Biomatrica) was used to dry quantitative DNA standard, primers and probes using a ratio of 1:4 DNA stable solution to sample volume. The solutions were mixed by pipetting every 2–5 min for 15 min and then dried in a DNA speed Vac – DNA110 (Savant). All samples were performed in triplicate. Samples were then rehydrated by adding the same volume of H_2_O as the original sample volume. The rehydrated samples were then tested in qRT-PCR using LightCycler 480 RNA master hydrolysis probes (Roche) as described above. Some samples were left in a dried condition for 2 weeks then rehydrated and tested by qRT-PCR to test long-term storage.

### 2.5 Real time RT-PCR

Primers and probe were adapted from Nilsen et al. (2007) and Madsen et al. (2009) using multiple phylogenetic approaches with different salmonid targets* (*STIM Scotland Ltd IP protected). The assays were designed for the α1a region (ATPase) of *S. salar*. The target region was ligated into plasmid pGEM3 and a quantitative DNA standard was derived as described by Weidmann et al. (2003).

#### 2.5.1 Laboratory qRT-PCR

For assay development NKA qRT-PCR was performed in 96 well skirted qPCR plates (20 μl) containing 1× LightCycler®480 RNA Master Hydrolysis Probes, 3.25 mM activator Mn(OAc)_2_, 500 nM primers, 200 nM probe and 1 μl plasmid DNA as template on an Agilent Technologies Stratagene MX3005P cycler (version 4.10, build 389). qRT-PCR reactions were ran in triplicate from 10^8^ to 10^1^ DNA molecules per reaction as follows: reverse transcription for 3 min at 63 °C, activation for 30 s at 95 °C, followed by 45 cycles consisting of amplification for 5 s at 95 °C and 15 s at 60 °C and a cooling step of 40 s at 40 °C.

For testing the samples of the S0 trials NKA qRT-PCR were conducted using Brilliant III ultra-Fast SYBR® Green qRT-PCR Master Mix (Agilent, Edinburgh, UK). Reactions were performed in a 20 μl volume containing 2× SYBR green qRT-PCR master mix, 500 nm primers and 1 μl RNA as template. RNA samples were grouped into I, M and F point plates and ran alongside two standard curves from 10^8^ to 10^1^ RNA molecules/reaction and 4 negative controls (NTCs) per plate. The temperature profile was adapted in the reverse transcription step for 10 min at 50 °C, activation for 3 min at 95 °C, followed by 45 PCR cycles as previously described.

#### 2.5.2 Mobile qRT-PCR

For transfer of the NKA qRT-PCR assay onto the mobile SmartCycler™ system, mobile qRT-PCRs were performed using the LightCycler 480 RNA master hydrolysis probes kit (Roche) in 25 μl SmartCycler™ tubes. Due to the increase in tube volume concentrations were kept the same as described above, but volume was increased for all components including template DNA (2.5 μl). Dried primer, probe and quantitative RNA standard were tested on the SmartCycler™ in triplicate to ensure sensitivity was not lost between devices.

### 2.6 Data collection and analysis

Unpaired *t*-tests were conducted on NKA qRT-PCR results and NKA activity assays for sites sampled at l and F testing points only. For sites sampled at I, M and F point's one-way ANOVAs were used. We compared copy number fold change (fc) between sampling time frames on sites for NKA qRT-PCR and NKA activity fc for NKA activity assays. Smolt indexes were taken for each fish at each site using a 3-point scoring system (Sigholt et al., 1995) based on silvering of fish, presence or lack of parr marks and changes in fin morphology. A number range from 1 to 4 was used, with 1 referring to parr like morphology and 4 to fully smoltified salmon morphology. Each score was totalled and averaged to give the final smolt index of the fish.

Average sea surface temperatures were obtained from the National Oceanic and Atmosphere Administration (NOAA) website using 1/4° Optimum Interpolation Sea Surface Temperature (daily OISST) data for 2015–2017 at 3 locations on the West coast of Scotland (South to North). Each fish farm was assigned one of the 3 location temperatures based on closest proximity. Daily temperatures between June and September during a 3-year period were analysed and data from each site were compared. Daily average temperatures were obtained from the Scottish Salmon company (SSC) at sites Russel burn and Geocrab for all 3 years of testing. Degree-days (dd) were calculated based on the formula dd = (^T^Max + ^T^Min/2) – T_0_ from temperatures recorded on site against a temperature threshold (T_0_) of 0 °C. ^T^Max and ^T^Min are the maximum and minimum daily ambient temperatures recorded. All I points for each site started at 0 dd with subsequent days calculated based on daily temperature records up until each F point. For sites that included M points these were also recorded. The data obtained was then analysed and compared between sites and years.

### 2.7 Mobile real time PCR

To test the NKA qRT-PCR onsite a suitcase laboratory was assembled based on experiences with a mobile laboratory developed for the detection of haemorrhagic fever viruses (Abd El Wahed et al., 2015; Faye et al., 2015). The mobile laboratory consisted of one suitcase containing everything needed for RNA extraction of gill arches, one suitcase containing a PCR flow workbench, and one suitcase containing the mobile SmartCycler™. A field trial was conducted on salmon smolts (*n* = 32) at H15. All extracted RNA was transported back to the Institute of Aquaculture on ice and re-tested on the SmartCycler™. To refine the mobility of the onsite NKA qRT-PCR platform as well as to validate it on an alternative PCR platform (Genesig Q16 qRT-PCR system), field trials were conducted at 3 hatcheries 9–11, H15 and H8, at two different time points. Gill arches were removed from 28 salmon smolts per site per visit (*n* = 28), and RNA extracted and tested using an ATPase kit (PrimerDesign Ltd) containing 2× Oasig master mix, dried primers and probe, positive RNA extraction control and positive ATPase control. The ATPase kit was developed using the same primers and probe used for the 3-year analysis and SmartCycler™ tests.

## 3 Results

### 3.1 NKA qRT-PCR assay development

**Fig. 2:**
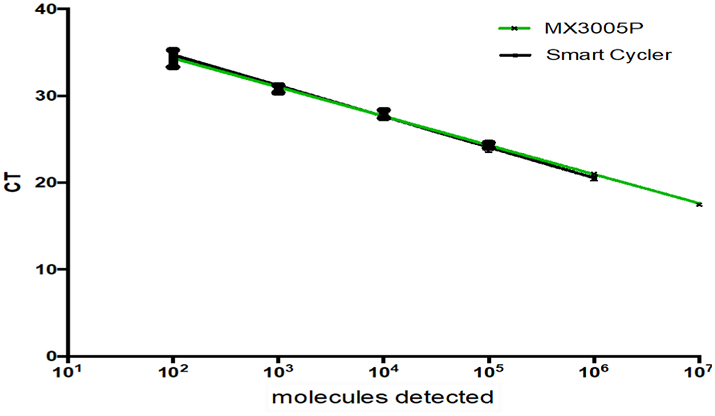
NKA qRT-PCR assay RNA standard. Green line: MX3005P (98.9%, SE 0.24), black: SmartCycler assay (93.43%, SE 0.119). Each dilution ranged from 10^7^ to 10^1^ RNA molecules. These were tested in triplicate with their mean C_T_ values plotted along with SE. (For interpretation of the references to colour in this figure legend, the reader is referred to the web version of this article.)

To validate primers and probes, qRT-PCR reactions were carried out in triplicates on the MX3005 cycler using a decimal dilution range of 10^7^ - 10^1^ RNA molecules per reaction. The NKA assay yielded a standard curve of high efficiency (98.9%, standard error (SE) 0.24) and sensitivity, detecting down to 10^2^ copies. The assay was successfully transferred to the SmartCycler™ system, tested in triplicate and yielded a robust standard curve showing high efficiency (93.43%, SE 0.119) (Fig. 2). The assay detected down to 10^2^ copies indicating no loss of sensitivity between devices. There was however a minor loss in efficiency of ~5%.

### 3.2 Dried primer, probe and standards tests

Primers, probe and standards were successfully dried and rehydrated after daily and long-term storage (2 weeks). Primers and probe were tested against both normal non-dried standard (10^5^) and rehydrated dry standard (10^5^) to check for any loss of efficiency or sensitivity. Both tests showed no loss of sensitivity to non-dried (C_T_ 24.3 ± 0.3) and dried standard, primer and probe (C_T_ 24.7 ± 0.6).

### 3.3 NKA activity assay validation in 3 year trial

**Fig. 3:**
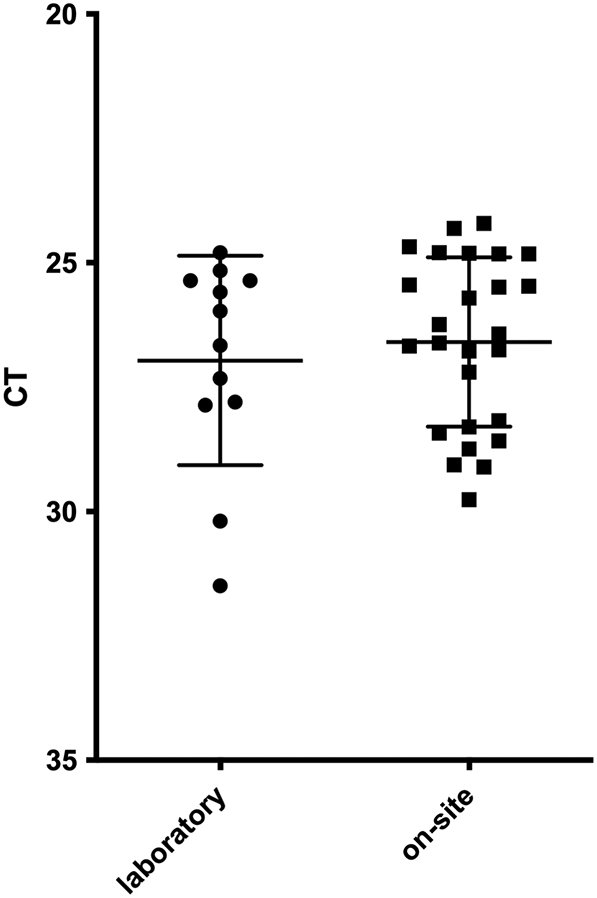
Comparison of NKA qRT-PCR results of samples from endpoint smolts of the same farm determined in the laboratory and on-site. Mean CT 26.96 (*n* = 12) for laboratory, and CT 26.59 (*n* = 26) for on-site results.

An initial comparison of assay performance in the laboratory and on site showed very good reproducibility (Fig. 3). A significant increase (*P* < 0.05) in NKA activity was observed in 12/16 hatcheries tested in 2015. The largest fold change (fc) was observed at H13 and 14 (tank A, 3.77; tank B, 3.7) while the lowest fc was recorded at H5 (1.19) In 2016 10/13 hatcheries sampled were found to have significant increases in NKA activity. The largest fc was found at H11 (3.73) and the lowest at H9 (1.41). One site showed a significant decrease in activity (H18 1.34). Six of nine hatcheries sampled in 2017 showed a significant increase in NKA activity and two hatcheries reporting results that were below the threshold of acceptable levels of NKA for safe transfer to SW (H13–14). The largest fc was at H17 (3.02) and the lowest at H6 and 7 (1.12). (Supplement figs. 1, 2, 3 and Table 1, Table 2, Table 3).

**Table 1:** Significance of change in NKA enzyme activity.

Year	Site	Initial vs Mid		Mid vs Final	
		Significance	*p*-value	Significance	*p*-value
2015	H13		0.663		0.966
	H9	*	0.046		0.723
	H10	*	0.006		0.987
	H11		0.162		0.978
	H12		0.331		0.09
	H8	*	<0.001	*	0.01
	H6		0.550		0.870
	H16	*	0.001	*	0.040
2016	H9	*	0.046		0.723
	H13		0.663		0.966
	H16	*	<0.001	*	0.040
	H8	*	<0.001	*	0.010
	H6		0.555		0.874
	H2	*	<0.001		0.866
2017	ORM		0.27	*	0.018
	H14	*	<0.001		0.997
	H6		0.443		0.095
	H1	*	0.037	*	0.034

Details of p-value and significance (*) for NKA activity change, I - M point and M - F points for 8, 6, 4 hatcheries sampled in 2015, 2016 and 2017.

**Table 2:** Significance of change in NKA RNA expression.

Year	Site	Initial vs Mid		Mid vs Final	
		Significance	*p*-value	Significance	*p*-value
2015	H13		0.096		0.058
	H9		0.443	*	0.042
	H10	*	0.043		0.733
	H11		0.539		0.338
	H12	*	<0.001		0.788
	H8	*	<0.001		0.999
	H6	*	0.028		0.403
	H16	*	0.001	*	0.040
2016	H9	*	0.011		0.455
	H13	*	<0.001		0.979
	H16	*	<0.001		0.860
	H8	*	<0.001		0.081
	H6		0.311		0.176
	H2	*	0.027	*	<0.001
2017	ORM		0.530	*	<0.001
	H14	*	<0.001		0.994
	H6	*	<0.001		0.209
	H1		0.421	*	0.017

Details of the mean Na^+^ K- qRT-PCR assays, p-value and significance (*) for I - M mid and M - F points for 8, 6, 4 hatcheries sampled in 2015, 2016 and 2017.

**Table 3:**
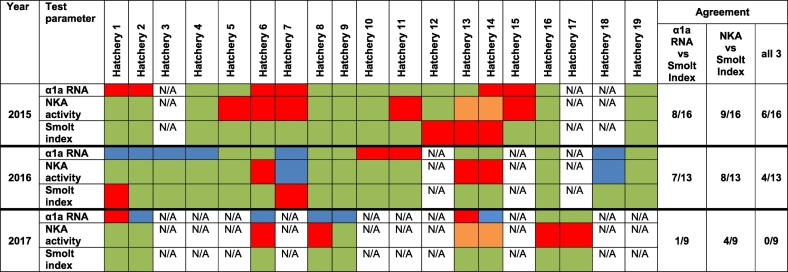
Comparison of NKA enzymatic activity and NKA α1a qRT-PCR expression to smolt index over the 3-year sampling period. It was found that in 2015 and 2016 both tests showed no significantly higher correlation with the smolt index. In 2017, NKA activity assays were shown to correlate higher (4/9) with smolt index than NKA qRT-PCR (1/9). Green tiles indicate significant increases for NKA activity assays and smolt index, and significant decrease in NKA qRT-PCR between I and F points. Red tiles show no significant change between I and F points. Blue tiles signify a decrease in expression of NKA activity assays and smolt index, and an increase in expression of NKA qRT-PCR. Orange tiles indicate where NKA activity assays did not reach the threshold of 10 for safe SW transfer.

When 16 hatcheries sampled in 2015 were analysed 4/8 sites showed a significant increase between I - M points and 2/8 between M - F points. H11 was the only hatchery to show no significance between all 3-time points. On analysis of the 13 hatcheries sampled in 2016 a significant increase between I - M points for 4/6 hatcheries and between M - F points for 2/6 hatcheries was observed. Only two hatcheries (H8 and H16) showed significant increases between I - M - F points. In 2017 (9 hatcheries sampled) NKA activity showed a significant increase between I - M points for 4/6 hatcheries analysed and for 2/6 hatcheries between M - F points. Again H8 and H16 showed significant increases between I - M - F points (Table 1).

### 3.4 NKA qRT-PCR assay validation in 3 year trial

In 2015 a significant decrease in NKA mRNA abundance was observed at 9/16 hatcheries. The largest significant decrease was found at H19 (3.26 fc) and the lowest at H13 (2.11 fc). Of the 6 remaining hatcheries, 4 showed no significant decrease in NKA copy number. H4 (2.75 fc) and H15 (1.67 fc) were the only tanks to show a significant increase in NKA copy number. H9–11 showed a universal decrease in NKA copy number over multi-tank sampling. A significant decrease was found in 7/13 hatcheries in 2016 with the largest fc at H13 (2.4), and the lowest at H19 (1.5). Four hatcheries showed significant increases in NKA copy number; H1 (2.66), H2 (1.3) and H3 (1.92), and H18 (1.53). Only one hatchery showed a decrease in NKA copy number between I - F points (H16) in 2017. Six hatcheries showed an increase in copy number between I - F points. The largest fc was at H9 (2.19) and lowest at H13 and H1 (1.1) (Supplement figs. 4, 5, 6 and tables 4, 5, 6).

When tested for significance 5/8 hatcheries analysed (16 hatcheries sampled) showed significant decreases of NKA mRNA expression between I – M points and 2 between M – F points for NKA mRNA in 2015. There was no correlation between individual sites with H6, 7, 9–11 sites showing opposite results for different tanks. H6 was the only hatcheries to show no change in NKA copy number over all 3 time points. In 2016, NKA mRNA expression showed a significant increase between I – M points for 5/6 hatcheries analysed (13 hatcheries sampled) and 1 hatchery for M – F points (H2), which also showed an overall decrease between I - M - F points. Of the 9 hatcheries sampled in 2017 NKA mRNA expression showed a significant increase between I – M points (H6, 7 and H14) and M-F (points (H1, 9–11) for 2/4 hatcheries (Table 2).

### 3.5 Sea water temperatures and degree days in 3 year trial

**Fig. 4:**
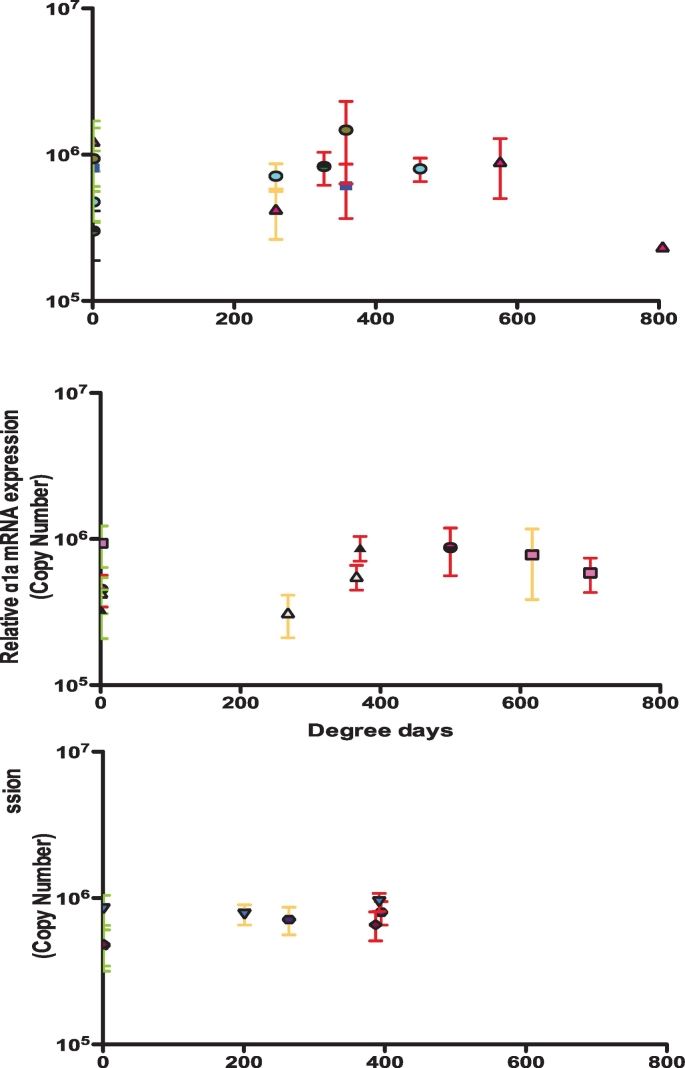
Mean NKA qRT-PCR mRNA copies expressed for I, M and F points for hatcheries (H) 1, 2, 3, 4, 6 and 7 against degree days (dd) over the 3-year study period. N per site at each time point = 8, NKA copy number expressed as mean ± SD.

Average sea temperatures for all 3 years were split into 3 sites based on closest location to each hatchery (Fig. 1). Site 1 consisted of H9–11 and 15. On average 2017 was warmer from Jun – Sep against 2015 (0.77–1.4 °C) and Jul – Aug against 2016 (0.1–0.69 °C). Site 2 included H1–7 and 16–17. On average 2017 was warmer than 2015 between Jun – Aug (1.12–1.79 °C) and 2016 between Jun – Aug (0.29–1.05 °C). Site 3 included H8, 12–14. Overall 2016 and 2017 were warmer on average than 2015 between Jun and September (0.41–1.57 °C) with 2016 and 2017 on average within 0.1–0.3 °C between Jun – Oct. The degree days (dd) between I - F points were fairly consistent for H1–5 across the 3-year study (327 – 392dd) as was the feeding regime period (FRP) (26–29 days) (Fig. 4). H3 2016 was the only site to have a longer FRP of 35 days resulting in 504dd at F point. For H6 and 7 there was a much higher variation in dd (255 – 706dd) and FRP (23–43 days). The shortest FRP and DD were observed at H7 2017 and longest at H6 2016. Five of the 14 hatcheries tested were observed to have FRP >400dd. This is past the generally accepted SW transfer window point.

### 3.6 Mobile NKA mRNA testing with mobile suitcase laboratory

**Fig. 5:**
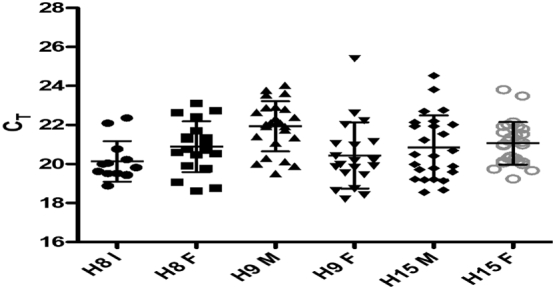
Onsite testing of the NKA qRT-PCR assay on the Q16 system at hatcheries (H) 8, 9 and 15. Tests were conducted at two different time points at 3 different sites. N per site at each time point = 28, NKA CT expressed as mean ± StD.

On site RNA extraction and mobile qRT-PCR analyses on the Q16 device were successfully carried out at all 3 sites (Fig. 5). There was a significant increase in NKA mRNA copy number at H9-11 between I – F points (*p* = 0.03). Both remaining sites showed no significant change in NKA copy number (H15: *p* = 0.59, H8: *p* = 0.08). There were notable issues with the positive control for both F points at H9-11 and H15. Positive control variation ranged from 13 to 17 C_T_ where a good positive control was expected to range from 17 ± 1.4 C_T_. Due to these issues we were unable to reliably compare copy numbers between sites.

## 4 Discussion

The aim of this study was to investigate if central laboratory based NKA enzyme activity testing could be replaced by real time RT-PCR with a potential to use a mobile real time RT-PCR directly on farm sites. Initially, a 3 year comparative assessment of both types of assay across 11 farm sites spread in a transect from south to north along the Scottish west coast was performed.

Analysis of the initial and final points across all three years showed a significant increase in NKA activity at 28/38 hatcheries tested and a significant decrease at 1 hatchery. The NKA activity threshold of 10 (as determined by STIM Scotland Ltd. based on historical data of successful transfers of fish) for SW readiness was not achieved in 8/38 hatcheries with 5 hatcheries showing SW readiness already at the initial point of testing. For real time qRT-PCR analyses NKA α1a mRNA expression significantly decreased at 17/38 hatcheries. In contrast, a significant increase was recorded at 7/38 hatcheries. The NKA activity assays results were comparable with the NKA RNA results at 15/38 hatcheries.

As a secondary reference these results were compared with the smolt index recorded at each site to try and better understand variations in the results of the two tests. For 2015 and 2016, both NKA activity assays and NKA qRT-PCR showed similar agreement with the smolt index, although not at all the same hatcheries (Table 3). In 2017 there was higher agreement between NKA activity assays and smolt indexes (4/9 hatcheries) than for NKA qRT-PCR (1/9 hatcheries). Overall, only 27% of all sites showed unanimous agreement between the 3 tests over the 3-year period.

### 4.1 Environmental factors obscuring smoltification assay results

Over this 3-year study one case of confirmed metal contamination was reported at Hatchery 1–2 and 4–5 in 2015. High levels of aluminium (Al) were detected at H1 and 2, with further low levels observed at H3–5. NKA α1a mRNA showed no significant decrease at H1 (1.57 fc) and 2 (1.32 fc), a significant increase at H4 (2.76 fc) and a significant decrease at H5 (2.5 fc). In contrast NKA activity assays indicated fish were ready for SW transfer (NKA activity >10) at H1 2.22 fc, H2 2.14 fc and H4 2.26 fc but remained stationary at H5 (1.1 fc). Smolt indexes signified a shift towards, but not complete smoltification, having increased significantly from the initial test points (H1 1.4–3, H2 1.6–3, H4 1.75–2.7 and H5 2.2–3.6). Degree-days were at or close to the appropriate SW transfer time of 350 - 400dd at H1–2 (358dd) and H4 (327dd) suggesting the fish were at the point of SW transfer. In contrast, saltwater bath tests were unsuccessful supporting the NKA qRT-PCR assay results. Transfer of these exposed fish back to FW resulted in an up regulation of NKA α1a mRNA and reduction in NKA α1b mRNA after 7 days, as would be expected in normal conditions, potentially as a compensatory mechanism (Richards et al., 2003; Nilsen et al., 2007; Madsen et al., 2009; Bystriansky and Schulte, 2011). The increase of α1a mRNA at the final point of H2 may have indicated that the fish had recovered from metal contamination and were back into full smoltification development (Nilsen et al., 2013). In general, the increase in α1a mRNA expression at final points at all four hatcheries may reflect fish health recovery.

All tanks required a further 3 weeks before fish were successfully transferred to sea suggesting the NKA qRT-PCR assay had indicated a failed smoltification process at H1, 2 and 4, in contrast to the NKA activity assays which had erroneously indicated a false positive signal for SW transfer at all 4 hatcheries. This is in line with previous research on metal and acid contamination indicating no effect on NKA activity (Monette and McCormick, 2008; Nilsen et al., 2013). There is counter evidence to this however indicating that moderate to high levels of aluminium and acidic water exposure can affect NKA activity (Scott and Sloman, 2004; Nilsen et al., 2010) and that the recovery period for smolts can be up to two weeks or longer (Nilsen et al., 2013).

Although no metal contamination was recorded in 2016 and 2017, due to the topography of the area it is possible low levels had been present, particularly during times of heavy rainfall. The hatcheries are located in a very rocky environment with many run off areas from the surrounding hills, high concentration of limestone (CaCO_3_), calcareous tufa, dolomite and colomite (CaMg(CO_3_) with an old disused copper (Cu^2+^) mine further upstream. Short periods of limited metal contamination could lead to small delays in smolt development than those shown in previous research into moderate and heavy contamination (Kroglund and Finstad, 2003; Kroglund et al., 2007, Kroglund et al., 2008; Monette and McCormick, 2008; Nilsen et al., 2010; Nilsen et al., 2013). Daily or weekly testing would need to have been carried out to validate or refute this. It should be taken into consideration however, that the dd recorded at F points for each of the 3-years were around the optimum time for SW transfer in regards to the literature (Stefansson et al., 1998; Handeland et al., 2004). At the H18 site, a decrease in NKA activity assay (1.34 fc) and an increase in NKA mRNA (1.53 fc), was observed, which could indicate the salmon were undergoing desmoltification. It is well documented that salmon smolts that remain in FW past the stage of full smolt development begin to regress morphologically back to a parr-like appearance whilst retaining all physiological changes (Folmar et al., 1982; Hoar, 1988). The smolt index for the initial point was relatively high compared to most sites (2.85), peaked at 3.5 at a mid point and remained similar at the final point (3.49). Fish going through desmoltification would be expected to have a very high smolt index for a prolonged period of time in FW before reverting back to their pre-smolt stage (Hoar, 1988) however the morphological appearance between smolt and desmoltified salmon has been shown to be relatively minor, with only small changes in skin pigmentation and body shape (Houde et al., 2019a; Houde et al., 2019b). Due to this it is not possible to assess whether desmoltification had occurred through smolt index observation.

The influence of temperature on smolt development is well documented (Solbakken et al., 1994; McCormick et al., 2000; Handeland et al., 2000, Handeland et al., 2004, Handeland et al., 2013). Smolts raised at higher temperatures (12–12.7 °C) will exhibit maximum levels of NKA activity 4 to 6 weeks earlier and thus advance the smolt process as opposed to those raised at lower temperatures (8.3–8.9 °C) (Handeland et al., 2000; Handeland et al., 2013). Morphological changes (parr marks, silvering of the scales) and an increase in body mass have been shown to develop faster in higher rearing temperatures in hatcheries (Handeland et al., 2004). Smolts maintained in FW past their SW transfer window (>400dd) at elevated temperatures (12–14 °C) showed significant downregulation of NKA activity after 4–6 weeks indicating desmoltification (Stefansson et al., 1998; Handeland et al., 2004). To ascertain whether temperature could have had some effect on smolt development the average sea temperatures for all 3 years were analysed and temperature profiles split into 3 sites based on closest location to each farm (Fig. 1). Although most temperature variations were quite minimal over the 3 years of testing the average temperatures for all years between July and September were above 12 °C and had maximums of 15 °C in some months for some years. When comparing dd between sites, it was found that H1–5 had kept within the optimal smolt transfer window (>400dd) (Stefansson et al., 1998; Handeland et al., 2004) at all but one of its F points (H3 2016, 504dd). H6 and 7 in contrast had only one site (H6 2017, 395dd) within this window with the remaining sites ranging from 460 – 700dd+. H1, 2, and 42,015, H1, 22,016 and H1, 2 and 62,017 were within the optimal smolt transfer window at F points and for all 3-years. NKA activity assays agreed with this as did smolt indices, showing a general move towards full smoltification characteristics in 5/7 hatcheries tested with optimal dd for SW transfer. In contrast, NKA qRT-PCR generally showed the opposite, with no significant change or an increase in copy number. Our analysis did not provide evidence for a correlation between temperature and smoltification and NKA activity or NKA expression results.

From the data gathered over this 3-year study, it would appear that there are two distinct drops in α1a expression, with an initial drop from the I to M phase of smoltification and a slower reduction in expression towards a baseline level of 1 × 10^5^ mRNA copies for SW ready smolts at the F phase. This is then followed by the rapid decrease in α1a down to low background levels in SW transferred smolts. A similar trend can be attributed to the NKA activity assays, with smolts reaching high levels of NKA activity near the M phase followed by a slow increases in NKA activity F phase SW ready smolts.

An in lab NKA qRT-PCR assay was successfully tested with samples from multiple fish farms over a 3-year period against NKA activity assays. For the first two years although not agreeing at all sites both assays had similar successes and failures in detecting smoltification (qRT-PCR 57%, NKA activity 60%).

The final year (2017) was the only variation to this, as all but one site exhibited down regulation of α1a mRNA by NKA qRT-PCR assay with NKA activity assays indicating successful smoltification at 4 sites. We were therefore not able to attribute any of the analysed factors as a contributor to assay performance.

### 4.2 Mobile testing of smoltification status on farm sites

A modified version of the mobile diagnostic kit containing less equipment was successfully tested. The extraction and assay were performed successfully at most sites with some issues recorded for internal positive controls, which limited our analysis. Positive control variations at Ormsary F and Uist F beyond ± CT 1.4 remained unexplained. We saw no significant decrease in ATPase C_T_ for all 3 sites, but an increase at Ormsary. This could be due to the varying time ranges of when we started sampling (mid smolt for Ormsary and Clachan), and the time between sample points (Loch Damph - 9 weeks, Clachan - 2 weeks and Ormsary - 4 weeks). In particular, Loch Damph smolts were to be held for another 2 weeks post final point sampling leading to a total smolt period of 11 weeks. Supersmolt® feed used to induce smoltification at this farm was only administered to fish after the second sampling indicating both were I points.

These experiences however also indicate that planning visits is crucial in applying the mobile qRT-PCR platform in order to efficiently test the I – M and F points reflecting the protocol and timeline implemented at each hatchery. The deployment study showed that we were able to optimise the suitcase lab by reducing its size and materials needed as well as optimise the RNA and mobile qRT-PCR protocols. The Q16 was also successfully able to run whilst being transported in a car, which opens up the possibilities for multiple site visits as tests from one farm can be conducted whilst travelling to the next.

### 4.3 Conclusion

Multiple qRT-PCR assays have been developed both academically and industrially for detection of ATPase α1a for in lab testing. We have successfully developed an in lab and a mobile qRT-PCR platform for the detection of the NKA transcript α1a. High sensitivity was shown for the in lab (98.9%, SE 0.24) and mobile (93.43%, SE 0.119) assays when tested using a quantitative RNA standard (both within optimal efficiency range of 90–105%). To our knowledge, this is the first assay developed in the lab that has been specifically modified to work on a mobile platform on site at fish farms. Both onsite assays were successfully tested on multiple farms. These tests have proven it is possible to test during a smolt period on site and provide results on the same day. This leaves the possibility for further refinement of the assay, development of other biomarkers and transferring viral assays targeting salmon disease for onsite testing.

## Abbreviations

**Table:** 

dd	degree days
fc	fold change
F-point	final-point
FRP	feeding regime period
FW	fresh water
GH	growth hormones
I-point	initial-point
M-point	mid-point
NKA	Sodium Potassium ATPase
SW	salt water

## Ethical approval and consent to participate

The study was approved by the Institute of Aquaculture Divisional Ethics Committee and Animal Welfare Ethical Review Body (15/16) 106.

## Consent for publications

All authors and STIM Scotland Ltd. have consented to the publication.

## Availability of data and materials

All data generated or analysed during this study are included in this published article [and its supplementary information files].

## Funding

This project was funded by 10.13039/501100000396TSB project TSB710523, SAIC project RD_2016_01 and BBSRC-Nerc Aquahealth project BB/M026183/1.

## Declaration of Competing Interest

NS. Was the CEO of Europharma UK at the time of the study. The authors declare that they have no known competing financial interests or personal relationships that could have appeared to influence the work reported in this paper.

## Appendix A Supplementary data

Supplementary material Image 1
