# Supplementary material for: Testing of NKA expression by mobile real time PCR is an efficient indicator of smoltification status of farmed Atlantic salmon
Source: Aquaculture. 2021 Nov 15;544:737085. doi: 10.1016/j.aquaculture.2021.737085 (PMC8386247; doi:10.1016/j.aquaculture.2021.737085)
Supplement: Supplementary file 1 — Supplementary material [file mmc1.docx]

**Supplement figures**

**Figure SF1.** Mean NKA activity assays for I, M and F points for 16 hatcheries (H) in Scotland (2015). N = 25, NKA enzymatic activity expressed as mean ± standard deviation (StD).

**Figure SF2**. Mean NKA qRT-PCR for I, M and F points for 16 hatcheries in Scotland (2015). N = 8, NKA copy number expressed as mean ± StD.

**Figure SF3.** Mean NKA activity assays for I, M and F points for 13 hatcheries in Scotland (2016). N = 25, NKA enzymatic activity expressed as mean ± StD.

F**igure SF4.** Mean NKA qRT-PCR for I, M and F points for 13 hatcheries in Scotland (2016). N = 8, NKA copy number expressed as ± StD.

**Figure SF5.** Mean NKA activity assays for I, M and F points at 9 hatcheries in Scotland (2017). N = 25, NKA enzymatic activity expressed as mean ± StD.

**Figure SF6.** Mean NKA qRT-PCR for I, M and F points for 9 hatcheries in Scotland (2017). N = 8, NKA copy number expressed as mean ± StD.

**Supplement Tables**

**ST1** Details of the mean NKA activity assays, fold change and p-value for initial and final points for 16 hatcheries sampled in 2015.

| Site | Initial Activity (Mean) | Final Activity (Mean) | Fold change | Significant (p<0.05) |
| --- | --- | --- | --- | --- |
| H13 | 1.97 ± 0.30 | 7.42 ± 3.60 | 3.77 | <0.001 |
| H14 | 2.29 ± 1.00 | 8.48 ± 2.44 | 3.70 | <0.001 |
| H8 | 6.06 ± 2.05 | 13.81 ± 3.74 | 2.27 | <0.001 |
| H4 | 7.91 ± 1.59 | 17.90 ± 4.90 | 2.26 | <0.001 |
| H1 | 6.71 ± 1.59 | 14.84 ± 3.43 | 2.22 | <0.001 |
| H9 | 6.50 ± 2.84 | 13.93 ± 3.21 | 2.14 | <0.001 |
| H2 | 6.02 ± 1.44 | 12.87 ± 3.37 | 2.14 | <0.001 |
| H10 | 6.99± 2.80 | 14.38 ± 3.85 | 2.05 | <0.001 |
| H12 | 6.98 ± 2.09 | 12.84 ± 4.10 | 1.84 | 0.004 |
| H16 | 6.28 ± 2.54 | 10.80 ± 4.54 | 1.71 | 0.032 |
| H6 | 9.92 ± 3.08 | 15.10 ± 3.93 | 1.52 | 0.064 |
| H11 | 6.97 ± 2.34 | 9.74 ± 3.12 | 1.39 | 0.113 |
| H15 | 7.60 ± 5.08 | 9.68 ± 2.76 | 1.27 | 0.331 |
| H7 | 11.00 ± 3.52 | 13.41 ± 2.46 | 1.21 | 0.1798 |
| H5 | 11.30 ± 2.50 | 13.50 ± 5.19 | 1.19 | 0.314 |
| H19 | 4.52 ± 1.936 | 13.38 ± 3.54 | 2.96 | <0.001 |
|  | | | | |

**ST2** Details of the mean NKA activity assays, fold change and p-value for initial and final points for 13 hatcheries sampled in 2016.

| Site | Initial Activity (Mean) | Final Activity (Mean) | Fold change | Significant (p<0.05) |
| --- | --- | --- | --- | --- |
| H13 | 6.08 ± 3.74 | 8.59 ± 3.31 | 1.41 | 0.511 |
| H14 | 3.95 ± 1.42 | 6.10 ± 1.81 | 1.54 | 0.020 |
| H9 | 7.83 ± 1.95 | 11.01 ± 2.16 | 1.41 | 0.008 |
| H10 | 6.10 ± 1.49 | 11.35 ± 2.93 | 1.86 | 0.002 |
| H11 | 4.00 ± 1.25 | 14.91 ± 2.73 | 3.73 | <0.001 |
| H16 | 4.13 ± 1.12 | 11.83 ± 3.17 | 2.86 | <0.001 |
| H18 | 13.31 +/ 2.10 | 9.92 ± 2.14 | 1.34 | 0.007 |
| H1 | 8.29 ± 3.69 | 14.33 ± 2.32 | 1.73 | 0.002 |
| H2 | 6.36 ± 2.45 | 14.42 ± 3.65 | 2.27 | <0.001 |
| H3 | 7.561 ± 3.08 | 17.93 ± 3.44 | 2.37 | <0.001 |
| H8 | 7.56 ± 1.10 | 13.70 ± 3.37 | 1.81 | <0.001 |
| H19 | 6.04 ± 1.50 | 19.11 ± 7.13 | 3.16 | 0.001 |
| H6 | 12.40 ± 1.93 | 13.33 ± 5.11 | 1.08 | 0.846 |

**Table ST3** Details of the mean NKA activity assays, fold change and p-value for initial and final points for 10 hatcheries sampled in 2017.

| Site | Initial Activity (Mean) | Final Activity (Mean) | Fold change | Significant (p<0.05) |
| --- | --- | --- | --- | --- |
| H8 | 11.36 ± 3.72 | 13.41 ± 1.65 | 1.18 | 0.177 |
| H9 | 6.99 ± 4.58 | 18.38 ± 4.92 | 2.60 | <0.001 |
| H13 | 3.16 ± 1.05 | 8.55 ± 2.43 | 2.70 | 0.001 |
| H14 | 3.63 ± 0.96 | 7.20 ± 1.71 | 1.98 | <0.001 |
| H6 | 12.11 ± 2.03 | 13.6 ± 3.96 | 1.12 | 0.612 |
| H1 | 5.52 ± 2.33 | 11.35 ± 2.15 | 2.06 | <0.001 |
| H2 | 8.15 ± 4.53 | 12.6 ± 2.30 | 1.55 | 0.026 |
| H17 | 5.15 ± 2.08 | 15.53 ± 1.23 | 3.02 | <0.001 |
| H16 | 5.54 ± 6.79 | 11.18 ± 6.75 | 2.02 | 0.118 |

**Table ST4** Details of the mean Na^+^ K- qRT-PCR assays, fold change and p-value for initial and final points for 16 hatcheries sampled in 2015.

| Site | Initial Copy number (Mean) | Final Copy Number (Mean) | Fold change | Significant (p<0.05) |
| --- | --- | --- | --- | --- |
| H19 | 2143000 ± 853292 | 656857 ± 449975 | 3.26 | <0.001 |
| H16 | 982143 ± 482088 | 337417 ± 131421 | 2.91 | 0.012 |
| H11 | 1458000 ± 508203 | 532857 ± 244397 | 2.74 | 0.001 |
| H9 | 1176000 ± 460046 | 399625 ± 191597 | 3.02 | <0.001 |
| H10 | 1319000 ± 844766 | 456375 ± 215721 | 2.89 | <0.001 |
| H8 | 2453000 ± 711874 | 670875 ± 295712 | 3.65 | <0.008 |
| H12 | 1917000 ± 667712 | 888375 ± 473250 | 2.16 | 0.001 |
| H13 | 1195000 ± 299151 | 567571 ± 313763 | 2.11 | <0.001 |
| H5 | 838375 ± 289400 | 334143 ± 226982 | 2.51 | 0.023 |
| H7 | 1502000 ± 762587 | 991333 ± 559937 | 1.51 | 0.220 |
| H2 | 809625 ± 248795 | 613250 ± 246717 | 1.32 | 0.135 |
| H6 | 1135000 ± 280587 | 846375 ± 514960 | 1.31 | 0.288 |
| H1 | 938125 ± 587866 | 1475000 ± 841963 | 1.57 | 0.164 |
| H14 | 230375 ± 52312 | 417875 ± 307338 | 1.81 | 0.130 |
| H15 | 607500 ± 636633 | 540125 ± 295695 | 1.12 | 0.791 |
| H4 | 300875 ± 111831 | 829333 ± 210599 | 2.76 | <0.001 |

**Table ST5** Details of the mean Na^+^ K- qRT-PCR assays, fold change and p-value for initial and final points for 13 hatcheries sampled in 2016.

| Site | Initial Copy number (Mean) | Final Copy Number (Mean) | Fold change | Significant (p<0.05) |
| --- | --- | --- | --- | --- |
| H13 | 1137000 ± 341164 | 476587 ± 197507 | 2.40 | <0.001 |
| H14 | 1148000 ± 414213 | 547688 ± 205257 | 2.10 | <0.001 |
| H9 | 1038000 ± 322637 | 618688 ± 137320 | 1.68 | <0.001 |
| H10 | 732563 ± 462127 | 691750 ± 199407 | 1.63 | 0.749 |
| H11 | 535375 ± 167498 | 449125 ± 191111 | 1.20 | 0.185 |
| H16 | 1373000 ± 246014 | 673000 ± 138819 | 2.04 | <0.001 |
| H18 | 736938 ± 137903 | 1128000 ± 201630 | 1.53 | <0.001 |
| H1 | 328938 ± 120884 | 875000 ± 168491 | 2.66 | <0.001 |
| H2 | 426636 ± 117626 | 553571 ± 106833 | 1.30 | 0.015 |
| H3 | 454000 ± 111541 | 874875 ± 314199 | 1.92 | <0.001 |
| H8 | 1264000 ± 337435 | 794188 ± 156557 | 1.59 | <0.001 |
| H19 | 775250 ± 205355 | 515533 ± 133851 | 1.50 | <0.001 |
| H6 | 935125 ± 296623 | 585375 ± 154943 | 1.60 | 0.005 |

**Table ST6** Details of the mean Na^+^ K- qRT-PCR assays, fold change and p-value for initial and final points for 9 hatcheries sampled in 2017.

| Site | Initial copy number (Mean) | Final copy number (Mean) | Fold change | Significant (p<0.05) |
| --- | --- | --- | --- | --- |
| H8 | 544125 ± 119475 | 852188 ± 148516 | 1.57 | <0.001 |
| H9 | 425467 ± 71487 | 929813 ± 259999 | 2.19 | <0.001 |
| H13 | 895375 ± 231684 | 812867 ± 169952 | 1.10 | 0.266 |
| H14 | 400938 ± 83463 | 641063 ± 152740 | 1.59 | <0.001 |
| H6 | 474938 ± 131467 | 800438 ± 145754 | 1.68 | <0.001 |
| H1 | 847500 ± 195862 | 945214 ± 132438 | 1.11 | 0.213 |
| H2 | 483357 ± 166367 | 657063 ± 147710 | 1.36 | 0.006 |
| H17 | 523462 ± 243882 | 686077 ± 169909 | 1.31 | 0.062 |
| H16 | 690563 ± 109574 | 525688 ± 204994 | 1.31 | 0.009 |
